# Accidental Oral Injuries by Electric Toothbrush: A Report of Three Cases

**DOI:** 10.1155/2020/8819850

**Published:** 2020-12-15

**Authors:** Miki Yamada, Shunsuke Hino, Satoshi Nakamura, Yosuke Iijima, Takahiro Kaneko, Norio Horie

**Affiliations:** Department of Oral and Maxillofacial Surgery, Saitama Medical Center, Saitama Medical University, Saitama, Japan

## Abstract

In recent years, electric toothbrushes have become widespread. However, injuries caused by electric toothbrushes have rarely been reported. We describe three cases of oral penetrating injuries caused by electric toothbrushes. Case 1 occurred in a disabled boy while brushing due to unexpected movement. In Case 2, a mother using an electric toothbrush had fallen when bumped by her child. Case 3 involved a man using the toothbrush while taking a bath, who slipped in the bathtub. Cases 1 and 3 were using sonic toothbrushes, and Case 2 was using an oscillating-rotating toothbrush. Electric toothbrushes can cause oral penetrating injuries and infections in the same manner as manual toothbrushes. Prevention of oral trauma requires familiarity with the form and function of electric toothbrushes. Some room for improvement remains in optimizing the form of electric toothbrushes.

## 1. Introduction

Intraoral injuries due to toothbrushes are common in children [1, 2]. Adults show a lower rate of toothbrush injury than children [3]. Most such injuries occur when the toothbrush is in the mouth and the patient falls or is knocked by another person [4]. In general, although toothbrush injury often involves penetration of the buccal mucosa or soft palate, recovery is achieved without serious complications, but in cases of deep penetration into the oral cavity, the toothbrush can reach the posterior neck and cause damage to important organs such as the trachea and large blood vessels, with potentially fatal results [5].

**Figure 1:**
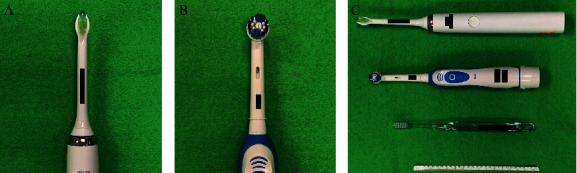
The two types of electric toothbrush. (a) Sonic toothbrush. (b) Oscillating-rotating toothbrush. (c) Electric toothbrushes and manual toothbrushes with scale.

Electric toothbrushes have also become popular in recent years, providing greater improvements in gingivitis and plaque removal compared to manual toothbrushes, improved ease of use, and also decreasing cost [6]. Two types of electric toothbrushes are available: sonic toothbrushes and oscillating-rotating toothbrushes (Figure 1). Sonic electric toothbrushes have a traditional brush head that moves side to side at high vibrational speed, while oscillating-rotating electric toothbrushes have a small, round brush head that rotates in one direction and then the other [7].

When brushing, oscillating-rotating toothbrushes have been considered safer than manual toothbrushes for surrounding hard and soft tissues, with the bristles contacting both teeth and gingivae [8]. Sonic toothbrushes have also been described as safe to use [9]. Injury caused by electric toothbrushes has been reported to account for 3% of all toothbrush-related injuries [2]. The most common injuries associated with the use of electric toothbrushes are reportedly eye injuries and injuries due to substances on the brush head (e.g., battery fluid) [2]. Additionally, breakages of electric toothbrushes due to product issues have been reported, resulting in damage such as broken teeth, choking, and swallowing of parts [10]. However, oral penetrating injuries due to electric toothbrushes have rarely been reported. Herein, we describe three cases of oral trauma caused by electric toothbrushes.

## 2. Case Report

### 2.1. Case 1

**Figure 2:**
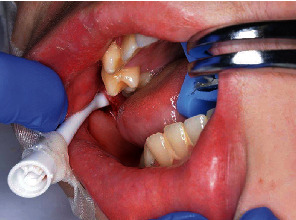
Case 1. The head of an electric toothbrush, taped by paramedics, is seen penetrating the right buccal mucosa.

**Figure 3:**
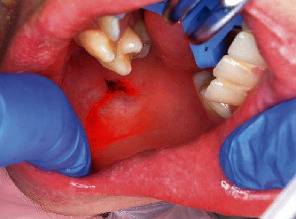
Case 1. The wound after removal of the head of the electric toothbrush.

**Figure 4:**
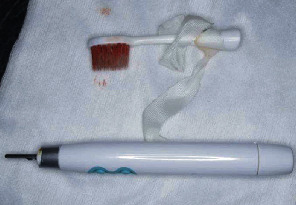
Case 1. The removed electric toothbrush, showing no apparent damage.

A 17-year-old boy was brought to the emergency room with penetration of an electric toothbrush into the buccal mucosa. The patient had Sturge-Weber syndrome (SWS) and quadriparesis. He was receiving tegretol at 350 mg/day, zonisamide at 370 mg/day, lamotrigine at 200 mg/day, sodium valproate 5% at 24 mL/day, levocarnitine at 4000 mg/20 mL/day, diazepam at 10 mg/day, and ramelteon at 8 mg/day. He received nourishment by nasogastric tube. When his father was performing daily oral care using an electric toothbrush in the evening, the head of the toothbrush penetrated the buccal mucosa when the patient moved unexpectedly. The father initially tried to remove the head, but could not. On examination, the head of the electric toothbrush had penetrated the right buccal mucosa (Figure 2). Facial angioma of the SWS was found in the right half of the face, including the upper lip, cheek, and orbital region. Under local anesthesia, the head of the toothbrush was carefully removed from the buccal mucosa with a small amount of bleeding (Figure 3). The injury site was rinsed adequately with saline, and suture was not performed because the tissue was considered to be contaminated. No damage to the head of the sonic electric toothbrush was evident (Figure 4). Amoxicillin hydrate and potassium clavulanate at 6060 mg/day were prescribed for 5 days. No complications were seen at follow-up after 1 week.

### 2.2. Case 2

**Figure 5:**
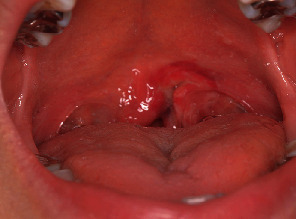
Case 2. Photograph about 15 h after the accident. The margin of the left soft palate is crushed.

A 38-year-old woman presented to the oral surgery clinic with an oral injury due to an electric toothbrush. The previous night, when she was brushing with an electric toothbrush, her child had bumped into her, causing her to fall. The toothbrush had penetrated the oral cavity, and bleeding from the posterior oral cavity was initially evident. The next day, the bleeding had stopped but the pain remained, so she visited the clinic. The patient was otherwise healthy and was not on any medications. Examination revealed a deep laceration on the left side of the posterior margin of the soft palate adjacent to the uvula and palatine tonsil (Figure 5). The margin of the wound was crushed, and although bleeding was minimal, severe pain was elicited on palpation. She had been using an oscillating-rotating electric toothbrush. As the toothbrush was intact and no symptoms suggestive of a serious condition were identified, no radiographic examination was performed. Clindamycin was prescribed at 600 mg/day for 5 days. Follow-up after 1 week showed no complications.

### 2.3. Case 3

A 67-year-old man visited the oral surgery clinic with swelling of the right cheek. While bathing during the previous night, the patient had started brushing with a sonic electric toothbrush. He then slipped in the bathtub and the toothbrush pierced the right buccal mucosa. He experienced a small amount of bleeding, but the toothbrush was not broken and the wound appeared small, so he went to sleep. The next morning, he noticed swelling of the cheek. He was a smoker with a history of 5 cigarettes/day, but no contributory medical history. On examination, the right cheek was swollen with a sensation of heat. A small, piercing entry wound that had already closed was recognized on the right buccal mucosa. Fluctuation was not evident. Serum examination showed a white blood cell count of 12.3 × 10^3^/*μ*L and a C-reactive protein level of 5.11 mg/dL, and intravenous drip infusion of ceftriaxone at 1 g/day was started (Table 1). On day 3, oral incision and drainage were performed. On day 7, the drain was removed. Antibiotics were then changed to oral amoxicillin at 750 mg/day. No complications were identified on follow-up after 1 week.

## 3. Discussion

Few reports appear to have described oral penetrating injuries caused by electric toothbrushes [2]. However, with the increasing adoption of electric toothbrushes, the number of reports is likely to increase in the near future.

The morphologies of the wounds were compared between the types of the electric toothbrush. Wounds with the sonic-type toothbrushes (Cases 1 and 3) showed small lacerations with smooth boundaries, similar to those from a manual toothbrush. This might be related to the fact that sonic and manual toothbrushes show similar shapes. Conversely, the wound from the oscillating-rotating type (Case 2) showed a jagged, piercing puncture wound. Although the site was the margin of the soft palate, which was originally fluttering, there was no denying that the rotating tufts of the brush head had damaged the soft tissue.

The treatment of electric toothbrush injury is the same as that for a long foreign body, including penetrating injury by a manual toothbrush [5, 11, 12]. With soft palate injuries, awareness of the potential for thrombosis and neurological complications due to carotid injury is necessary. In such cases, 72 h of follow-up is required [12]. In cases of toothbrush-associated injury, since the toothbrush is contaminated with oral bacteria, care must be taken to prevent infection [13]. In cases of electric toothbrush injury, the moving and vibrating bristles can spread bacteria in the tissue further than manual toothbrushes, regardless of the type. In Case 3, the wound was already closed before consultation, and the infection appeared to be progressing with abscess formation, given findings such as the sensation of heat and the elevated white blood cell count.

The reason toothbrush injury is less common in adults than in children is most likely that adults are less likely to fall. The major difference in shape between a manual toothbrush and an electric toothbrush is that the electric toothbrush typically has a longer handle. Of the present three cases, Cases 2 and 3 might not have penetrated as deeply if the handle had been shorter. This suggests that if a child falls while using an electric toothbrush, tissue penetration might tend to be deeper and the consequences could be more serious. Case 1 occurred during brushing being performed for a disabled boy. Recently, electric toothbrushes have also seen increasing use for brushing in individuals with limitations on activities of daily living and have shown effectiveness [14]. While electric toothbrushes are convenient, injury-prevention strategies require recognition of the differences in form and method from manual toothbrushes.

It was suggested that the following four strategies appear important for preventing accidental oral injuries caused by manual and electric toothbrushes: no brushing while taking a bath or in similar situations where the user is at heightened risk of slipping or falling; no brushing while walking in the house or concentrating on another activity; while brushing the teeth of a child, their head should be stabilized to prevent unexpected movements; and use of a manual toothbrush should be considered for brushing the teeth of a disabled individual, particularly if the individual is prone to making sudden and unexpected movements. Furthermore, regarding the form of the toothbrush to reduce accidental oral injuries, the following three recommendations were identified: shortening the electric toothbrush handle for children, since the distance to the oropharynx is shorter; making the handle of the electric toothbrush from a flexible material that might soften any injury; and using markers on the toothbrush handle that can indicate to the user the distance the toothbrush can be placed inside the mouth (similar to the orange filter on a dental light cure).

In conclusion, electric toothbrushes are convenient, but, like manual toothbrushes, can cause penetrating injuries and infections and sometimes may result in more serious injuries. Preventing oral trauma requires familiarity with the form and function of the electric toothbrush. Various improvements in the form of electric toothbrushes may still be worth adopting.

## Figures and Tables

**Table 1:** Laboratory data and clinical course of Case 3.

	Day 1	Day 2	Day 3	Day 4	Day 5	Day 6	Day 7	Days 8–10
WBC (×10^3^/*μ*L)	12.3				6.0		5.9	
CRP (mg/dL)	5.11				4.06		1.70	
Antibiotic	CTRX	CTRX	CTRX	CTRX	CTRX	CTRX	CTRX	AMPC
Treatment			Drainage				Removal of drain	

WBC: white blood cells; CRP: C-reactive protein; CTRX: ceftriaxone; AMPC: amoxicillin. Normal ranges of WBC and CRP are 3.25 to 8.59 (×10^3^/*μ*L) and 0.00 to 0.30 (mg/dL), respectively.
